# Effect of canal blocking on biodiversity of degraded peatlands: Insight from West Kalimantan

**DOI:** 10.1371/journal.pone.0334014

**Published:** 2025-10-08

**Authors:** Muhammad Ali Imron, Farah Dini Rachmawati, Tungga Dewi, Dennis Albihad, Giot Marganti Ito Simanullang, Erin E. Poor

**Affiliations:** 1 Faculty of Forestry, Universitas Gadjah Mada, Jalan Agro Bulaksumur, Yogyakarta, Indonesia; 2 WWF Indonesia, Forest and Wildlife Directorate, Jalan TB Simatupang Kv 38, Jakarta Selatan, Indonesia; 3 Lembaga JAWI Indonesia, Gedongkiwo MJ.I/848, Mantrijeron, Yogyakarta, Yogyakarta Special Region, Indonesia; 4 Tackle Climate Change, The Nature Conservancy, North Fairfax Drive, Suite 100, Arlington, Virginia, United States of America; National Research and Innovation Agency, INDONESIA

## Abstract

Large-scale disturbance in peatland areas causes many wildlife species to suffer due to limited resources or habitat loss. Following the high attention to peatlands, some restoration efforts, such as canal blocking, have been undertaken to restore the hydrological condition of peatlands. Nonetheless, our understanding of peatland biodiversity post-canal blocking is still limited. Thus, we conducted a study in West Kalimantan to assess wildlife diversity in peatland rewetting areas and understand the canal blocking’s impact on peatland biodiversity. Surveys were conducted during both the wet and dry seasons using line transects and point counts, along with deploying autonomous recording units in four habitat types: less disturbed peat forest, disturbed peat forest, estate crop, and disturbed wet shrub. Shannon and Simpson diversity indices suggest that habitats with complex structures (i.e., forested habitat) support higher diversity compared to those with open and uniform vegetation (i.e., non-forested habitat). This notion is also supported by acoustic indices calculations, which indicate that forested areas show higher acoustic diversity (biophony), and thus higher biodiversity compared to non-forested areas. However, our results indicate that there is insufficient evidence to suggest the effect of canal blocking on wildlife diversity in all rewetting sites. Aligning biodiversity conservation efforts with the natural climate solutions hierarchy, which is part of nature-based solutions and consists of protect, manage, and restore, could offer promising solutions for recovering the sites.

## Introduction

Global peatlands may only hold three per cent of global land, yet it stores approximately 25% of the world’s soil carbon [1,2], and contribute a critical role in greenhouse gas (GHG) sequestration; thus, climate change mitigation [3]. However, due to the large scale of disturbances in peatland areas such as logging, forest clearance, and industrial plantations, instead of yielding benefits, peatlands may generate catastrophes. Converting peatlands into cultivation by draining peat water through canal building and clearing the land using the burning method, as well as logging, can lead to more carbon emissions, increased wildfire regimes, severe health problems, and loss of biodiversity [4–11]. Tropical peatlands are unique and essential ecosystem holding many values towards human livelihoods and surrounding environments. The hydrological system allows peat water to be the source and supply of adjacent rivers and regulates the global and local water cycle, thus alleviating droughts and floods [12].

Indonesia holds 36% of the global area of peatland, with 485.6 ± 3.2 MtCO_2_e yr − ^1^ in carbon stocks [13,14]. However, many peatlands in Indonesia are threatened by land conversion, which also puts biodiversity throughout the tropics at risk [15,16]. In the Giam Siak Kecil-Bukit Batu Biosphere Reserve, the conversion of tropical peatland into acacia and rubber plantations has resulted in lower bird diversity compared to natural peat forests [17]. Following a peat fire in Teluk Meranti, there has been a significant alteration and reduction in tree species diversity compared to the unburnt forest [18]. In Kalimantan, the peatland fire of 2015 had repercussions on the behavior and survival rates of wildlife in both terrestrial and aquatic ecosystems [19]. Therefore, the impact of peatland degradation on biodiversity cannot be overlooked, especially considering recent long-term research findings and the increasing interest in biodiversity surveys which have revealed that tropical peatlands are home to diverse wildlife with few yet essential ecosystem endemic species [9,20–25].

With increasing global awareness of the importance of natural systems contributions to the global climate crisis, the peatland crisis has gained much attention not only from scientists but also from the government [26,27]. In response to the damaging 2015 peatland fires across Indonesia, the Indonesian government showed its commitment to peatland restoration by establishing the Peatland Restoration Agency in 2016 (now known as Badan Restorasi Gambut dan Mangrove), mandated to accelerate peatland restoration. Several programs have been conducted to restore the degraded peatland, primarily through rewetting by raising groundwater levels, revegetation by establishing vegetation cover, and revitalization by empowering local communities through sustainable livelihood programs [28]. While the progress and outputs of rewetting, revegetation, and revitalization (e.g., groundwater level, vegetation cover, seed survival, sustainable local businesses) are subjects of growing study, how biodiversity responds to these developments is still poorly understood [6,29–35].

However, given the pre-restoration disturbances, it is assumed that most wildlife species suffered due to scarce resources, isolation, or complete habitat loss [34,36]. Little research has been conducted on biodiversity in degraded peatlands and the research that exists is limited to just a few locations [25,37,38]. It is often assumed that survived or escaped wildlife will likely migrate back when the degraded habitat recovers [39–44]. In the long term, that returned wildlife could help restoration programs in peatlands by acting as seed dispersal and taking part in nutrient flux, thus boosting natural regeneration [45–49]. Therefore, it is necessary to further understand wildlife diversity in restored peatland areas to monitor the program’s impact on peatland biodiversity restoration. This paper will study the state of biodiversity in rewetting sites in the Mempawah and Kubu Raya Landscapes, West Kalimantan. Comparing wildlife diversity across various rewetting sites can provide insights into how animal species respond to canal blocking, a method used to restore the hydrological condition of peatlands. Here, we aim to conduct biodiversity surveys in four different types of rewetting sites: less disturbed peat forest, disturbed peat forest, estate crop, and disturbed wet shrub.

Our study integrated two types of wildlife surveys to optimize time and enhance our understanding of wildlife presence in rewetting sites in West Kalimantan, Indonesia. Direct detection by humans via walking points and transects aimed to create a species presence list at each surveyed site. Additionally, we deployed automatic recording units (ARUs) to record soundscapes throughout the day and night (referred to as passive acoustic monitoring or PAM), providing a more comprehensive view of wildlife activity. With this study, we also aimed to understand further biodiversity by assessing habitat features. In the end, we seek to explain the state of biodiversity in each rewetting areas and paralleling it with management implications.

## Materials and methods

### Study site

**Fig 1:**
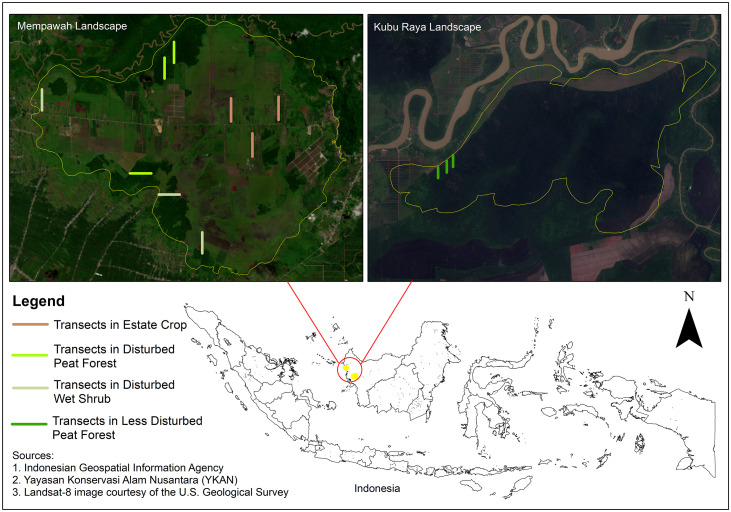
Site map pointing survey location in landscape of West Kalimantan.

**Fig 2:**
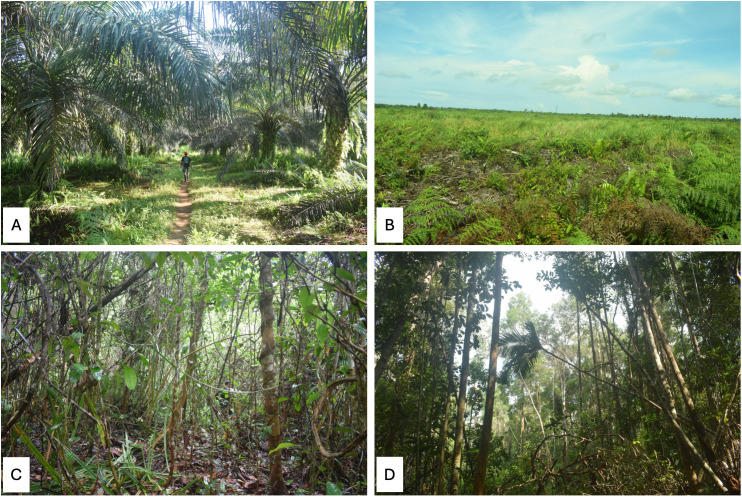
Habitat preview with figure. (A) showing estate crop, (B) showing disturbed wet shrub, (C) showing disturbed peat forest, (D) showing less disturbed peat forest.

Our focus is on examining peatlands in West Kalimantan, Indonesia (Fig 1). We assess habitat features and biodiversity at two landscapes: Mempawah and Kubu Raya. Mempawah consists of three habitat types: disturbed peat forest, estate crop, and disturbed wet shrub, whereas our study site for less disturbed peat forest is located within a community forest reserve in Kubu Raya landscape (Fig 2). Each site has its own characteristic, which we compile in Table 1. We conducted the field surveys twice, once during the wet season and the other during the dry season. The initial survey was conducted from May 5th to May 26th, 2023, to collect data during the wet season, followed by a dry season survey from August 3rd to August 25th, 2023.

**Table 1:** Characteristics of each study sites.

Landscape	Habitat type	Site information
Mempawah	Disturbed peat forest	• Located in two villages: Sungai Bakau Besar Darat and Sejegi • Still actively logged by the local people to collect pilling wood • Surrounded by palm oil plantation and wet shrub • Canal blocking was established in 2017 and 2018
	Estate Crop	• Located in Anjungan Dalam Village • Consist of palm oil plantation owned by individuals and corporations • Understory cover is often low due to intensive management; however, in some spots, we also found areas with dense understory cover. • Near crossroad • Canal blocking was built in 2018
	Disturbed wet shrub	• Located in three villages, i.e., Sungai Bakau Besar Darat, Sungai Rasau, and Antibar • Wet shrub in Antibar is actively managed for agriculture (pineapple and herb plantation) • Wet shrub in Sungai Bakau Besar Darat bordered with secondary peat forest • Wet shrub in Sungai Rasau is located near land clearing for oil palm plantations and has experienced repeated fires • Canal blocking was built in 2015, 2017, and 2018
Kubu Raya	Less disturbed peat forest	• Located in Permata Village • The forest is community forest reserve. • Rarely entered by people • Experienced low disturbance since logging activity stopped approximately 10 years ago • Bordered with palm oil plantations • Canal blocking near the forest was established in 2021

Prior to data collection, we obtained approval to conduct the study from the Dean of the Faculty of Forestry, Universitas Gadjah Mada. As the study was conducted across two regencies and involved lands owned by the village and individual community members, we were required to obtain permissions from both provincial and local authorities. We received written approval from the West Kalimantan Province Office of Investment and Integrated One-Stop Services (DPMPTSP Kalimantan Barat), as well as verbal permission from the head administrators of each village where the research took place.

### Survey methods

#### Wildlife attributes.

We collected wildlife data by utilizing line-transects to assess mammals and herpetofauna. We established three line transects within every habitat category, resulting in a total of 12 transects (Fig 1). Each transect had a length of 1 km and was positioned perpendicular to the neighboring access road or canal. The starting point of each transect was set at 100 meters from the road or canal to minimize edge effects. Upon sighting a species, we recorded species name, number of individuals, and coordinates.

We used point counts to assess bird diversity. Along the transects, six point-counts were established, each with a circle radius of 50 m, resulting in a total of 72 points. To prevent double counting during observation, there was a 200 m spacing between each point count. Each point count was assigned a 10-minute observation window. During the observation period, data including species name and individual count were recorded. Bird positions were documented using the coordinates of the point location.

To collect soundscape data, we deployed a set of SwiftOne (The Cornell Lab of Ornithology K. Lisa Yang Center for Conservation Bioacoustics) passive acoustic recorders across four habitat types. Each recorder was systematically positioned at the beginning and end of each transect, affixed to tree trunks at approximately 130 cm off the ground. Each device was set at minimum 500 m apart to ensure acoustics sampling were independent [50,51]. The acoustic devices were programmed to continuously capture sounds for 59 minutes every hour over a span of three days (equivalent to 3 x 24 hours), synchronized with the visual surveys conducted by human observers. Sound data collection occurred consistently in May and August, employing uniform tool settings and recorder placement.

#### Habitat features.

1. Vegetation

We collected vegetation data using nested plots, each with a specific plot size designated for different vegetation and tree growth stages. We used 1x1 m plots to observe understory which includes non-woody plant species, 2x2 m plots to observe woody seedlings, 5x5 m plots to observe woody saplings (those with diameter less than 10 cm), 10x10 m to observe woody pole (diameter ranging from 10–20 cm), and 20x20 m plots to observe tree (i.e., tree with diameter exceeding 20 cm). These nested plots were systematically placed within a transect, with each transect containing six plots spaced 200 meters apart. During each observation, we recorded essential vegetation attributes, including plant species, individual counts, and tree height and diameter (specifically for saplings, poles, and mature trees). Additionally, we measured canopy cover and ground cover using HabitApp.

1. Environmental data

We used data loggers (Mini USB, model BTH81, Bonajay Technology Co., Ltd.) to document local temperature and humidity were also placed in the same spot as acoustic recorders. We set the loggers to note the information every 5 minutes continuously throughout 24 hours. Effective total duration in data logging was also set to coordinate with PAM as well as human survey. Environmental data collection was done in 6–25 May 2023 and 4 to 24 August 2023 using the same tools setting and placement.

### Data analysis

#### Wildlife attributes.

We gathered data on both individual counts and species richness to assess biodiversity within different habitats. Various indices were computed based on this data to evaluate biodiversity levels. Menhinick’s and Margalef’s indices were used to determine species richness, while Pielou’s Evenness Index measured evenness across habitats and the Berger Parker Index assessed dominance. Additionally, the Shannon and Simpson Indices provided insights into diversity levels within specific areas. After obtaining index values, we compared them using one-way ANOVA for normally distributed data and Kruskal-Wallis tests for non-normally distributed data. Prior to comparisons, we tested for normality using the Shapiro-Wilk test. Subsequently, we conducted post-hoc tests using Tukey’s honestly significant difference (HSD) to identify specific groups with significant differences following the detection of overall mean variations.

We utilized Generalized Linear Model (GLM) to identify relationships between each index and habitat parameters. Here, the indices served as dependent variables, while habitat parameters (including temperature, humidity, canopy cover, ground cover, tree density, pole density, sapling density, seedling density, understory density, vegetation richness, month, and habitat type) acted as independent variables. Model selection was guided by comparing the Akaike Information Criterion (AIC) values, with lower AIC values indicating better model fit. We systematically eliminated independent variables that did not significantly affect the dependent variables, as indicated by high p-values, to produce the best regression model with the lowest AIC.

To identify wildlife from acoustic data, we calculated acoustic indices, i.e., biodiversity calculation based on acoustic cues [52], using Kaleidoscope Pro 5.6.2 software (Wildlife Acoustics, USA). All audio recordings retrieved from the first and second field surveys were used without any filtering or special selection. We selected four acoustic indices as proxies to quantify biodiversity: Acoustic Complexity Index (ACI), Acoustic Diversity Index (ADI), Acoustic Evenness Index (AEI), and Biodiversity Index (BI). An increased value in ACI, ADI, and BI can be understood as a rise in biological activity, which may further suggest greater biological diversity. The AEI value needs to be carefully interpreted, as a lower number suggests a more even distribution of sound energy across all frequency bands, indicating reduced dominance by a single species or a few species. The numbering of acoustic parameters followed the default settings from Kaleidoscope Pro.

Statistical analyses were done in Rstudio 2023.06.2 + 561 (Posit Software, PBC). Bio-acoustic data were fitted into a Linear Mixed Model (LMM) using the ‘lmer’ function from the ‘lme4’ package (Bates, 2015). The Y variables were acoustic indices and macro taxa detections. Firstly, we included month, transect code, and transect position as a nested random factor, with habitat as a predictor. We ran an ANOVA (type II analysis of variance) from the ‘car’ package [53] to observe the differences in Y variables between habitat types. Pairwise comparisons of two habitats were conducted using the emmeans test from the ‘emmeans’ package [54].

We ran a second linear mixed model, which included environmental variables (temperature) and vegetation features (canopy cover, ground cover, number of plant individuals, and Shannon diversity of plants) as fixed factors. Acoustic indices were the Y factors, while the nested random factors comprised month, habitat, transect position, and transect code. Predictors were selected based on multicollinearity tests and VIF values below 3. We set all p-values significant at 0.05 or a 95% confidence interval.

#### Habitat features.

To visualize plant community compositions across different habitats, we employed a Non-Metric Multidimensional Scaling (NMDS) using the vegan package in R [55]. We applied metaMDS function utilizing Bray-Curtis dissimilarity. The NMDS analysis yields a stress value of 0.112, indicating an adequate fit to the original dissimilarity data. In addition, we presented temperatures and humidity data for all habitats using boxplots to provide an overview about the data distribution.

## Results

### Wildlife attributes

Species richness was notably higher in August compared to May, with 80 species observed in May and 89 species in August across all habitats (Table 2). In both surveys, disturbed peat forest had the highest species count, while estate crop had the lowest. Examining the distribution of data from each transect, less disturbed peat forest and disturbed peat forest consistently exhibited a wider range of species richness compared to estate crop and disturbed wet shrub. Moreover, based on the index calculations, Menhinick’s and Margalef’s Richness Indices indicated lower species richness in May, which increased in August across all habitats. Overall, both indices highlighted that less disturbed peat forest and disturbed peat forest had greater species richness compared to estate crop and disturbed wet shrub.

**Table 2:** Wildlife attributes at each habitat type.

Indices	1st Survey (May)				2nd Survey (August)			
	LF	DF	EC	DW	LF	DF	EC	DW
Number of Individuals	155	146	190	184	128	135	149	131
Species Richness	37	40	28	35	39	40	27	33
Menhinick’s Species Richness	2.97	3.31	1.96	2.58	3.45	3.44	2.13	2.88
Margalef’s Species Richness	7.14	7.83	5.15	6.52	7.83	7.95	5.20	6.56
Pielou’s Evennes	0.90	0.87	0.81	0.87	0.89	0.90	0.78	0.79
Berger Parker Domince	0.11	0.18	0.29	0.14	0.12	0.10	0.33	0.25
Shannon Diversity	3.23	3.19	2.67	3.11	3.25	3.33	2.53	2.76
Simpson Diversity	0.95	0.94	0.89	0.94	0.95	0.96	0.86	0.89

LF: less disturbed forest, DF: disturbed forest, EC: estate crop, and DS: disturbed wet shrub.

Pielou’s Evenness Index, ranging from 0 to 1, indicates the level of evenness within a habitat, with higher values signifying greater evenness. In general, forested habitat are recognized for their higher evenness compared to estate crop and disturbed wet shrub. The elevated evenness index values in forested habitats suggest a lack of dominance by any single species, aligning with the findings of the Berger Parker Dominance Index. This index, varying from 0 to 1, reflects dominance, with larger values indicating greater dominance. Analysis reveals that estate crop is dominated by a few species in both months, while the opposite occurs in less disturbed peat forest which consistently had lower dominance. Although disturbed wet shrub exhibited low dominance values in May, these values increased in August, while disturbed peat forest displayed a decrease in dominance over the same period.

We used the Shannon and Simpson Diversity Indices across all four habitats. Overall, forested habitats exhibited greater diversity indices compared to non-forested habitats. Less disturbed and disturbed peat forests consistently maintained Shannon Diversity values above 3 in both May and August, while disturbed wet shrub displayed values of 3.11 in May, dropping to 2.76 in August. Estate crop consistently exhibited Shannon Diversity Index values below 3 in both months. There were distinct trends observed in Shannon Diversity Index values: forested habitats experienced an increase in values after the August resurvey, whereas non-forested habitats exhibited a decline. Similar trends were observed in Simpson Diversity Index values, with forested habitats maintaining consistent values while estate crop and disturbed wet shrub exhibited lower values in August compared to May.

**Fig 3:**
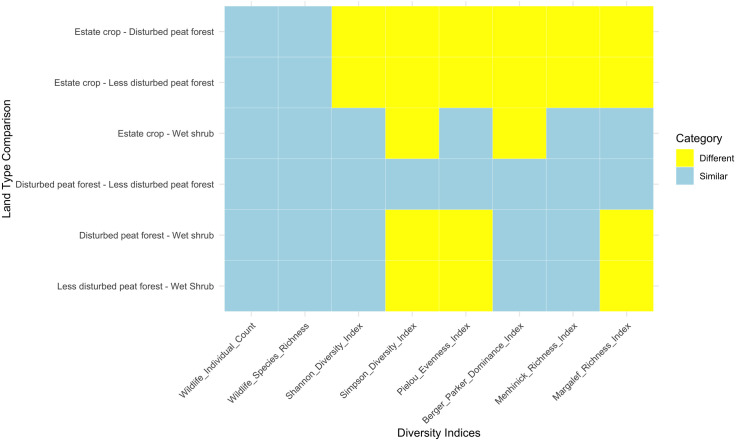
Post-hoc tests using Tukey’s Honestly Significant Difference (HSD) for wildlife attributes.

Following the computation of indices for each habitat, we conducted a comprehensive comparison test to ascertain significant differences between them. The p-values associated with wildlife individual count and species richness indicated no significant differences among the habitats. However, other indices such as Menhinick’s Species Richness, Margalef’s Species Richness Pielou’s Evennes, Berger Parker Dominance, Shannon Diversity, and Simpson Diversity indicated statistically significant variations. Post-hoc analysis revealed that less disturbed and disturbed peat forests exhibited similarities across these indices, contrasting with estate crop, which demonstrated differences in these indices compared to forested habitats (Fig 3). Additionally, disturbed wet shrub displayed discrepancies from forested habitats across several indices, including Simpson’s Diversity Index, Pielou’s Evenness Index, and Margalef’s Richness Index.

**Fig 4:**
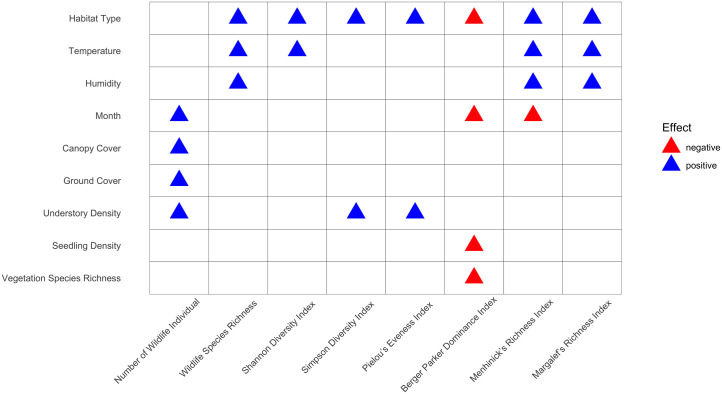
GLM result of wildlife attributes. Column showing predictors while rows showing explanatory factors.

According to the results from the GLM test, only 9 out of 12 habitat variables demonstrated linear relationships with the indices, with habitat type significantly influencing most response variables, indicating its substantial impact on determining index values in each habitat (Fig 4). Additionally, variables such as temperature, humidity, month, and understory density also exerted notable impacts on the indices, with nearly all response variables showing a positive relationship with the predictors, except for the Berger Parker Dominance Index, which exhibited a significant negative effect. Furthermore, a comparison test was conducted for habitat parameters linearly related to the indices to assess differences in environmental conditions across habitats. Six variables exhibited statistically significant differences (p > 0.05), including temperature, humidity, seedling density, understory density, canopy cover, and vegetation species richness, whereas ground cover remained consistent across habitats (p < 0.05), suggesting no significant variation between them.

The consistency pattern between forested and non-forested was also detected within acoustics indices. Although it may not be specific to one habitat type, but for ADI and BI the highest value was noticed in forested areas and lowest in non-forested areas (S1 Fig). As for AEI, highest value was in non-forested areas and lower in forested areas. Meanwhile for ACI, less disturbed peat forest holds the lowest value and highest in disturbed wet shrubs. Within month comparison, we identified change from mean value May to August from ADI and AEI where in all habitat types of ADI increased and AEI decreased (S3 Table). Mean BI value increased from May to August in all habitat types except estate crop. Mean ACI increased in disturbed wet shrubs and disturbed peat forest while decreasing in estate crop and less disturbed peat forest.

**Fig 5:**
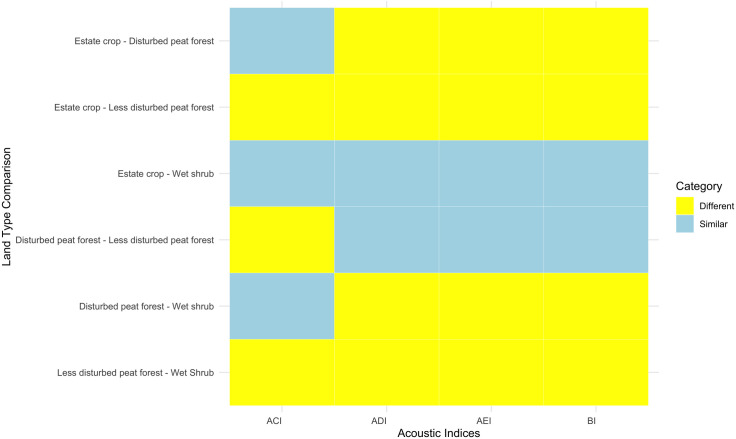
Pairwise results from LMM of acoustic indices.

We used LMM to test differences between habitat types. ACI, ADI, AEI, BI were placed as predictors. Habitat was placed as fixed variables, whereas month, recorder position (start and end of the transect), and transect code were set as random variables. Initial Type II ANOVA results from the car package showed significant p-value for ACI, ADI, AEI, and BI, indicating differences between habitats (S9 Table). The following pairwise test showed that forest and non-forest pattern was apparent across bioacoustic factors (Fig 5). The value of ADI, AEI, and BI in disturbed peat forest has similarity with less disturbed peat forest, while disturbed wet shrub similar with estate crop. In addition, those third acoustic indices in forested habitat show differences with non-forested habitat.

**Fig 6:**
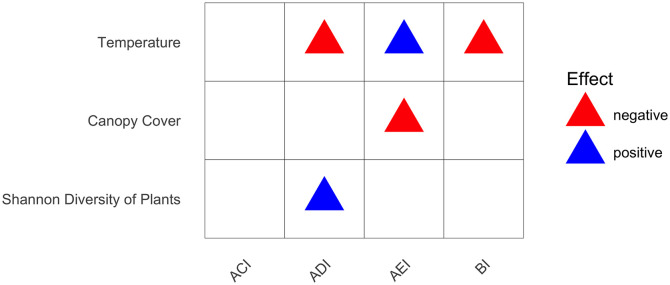
Linear mixed model with acoustic indices as predictors and rows as explanatory variables.

For the regression model, fixed effects included temperature, canopy cover, ground cover, number of tree individuals, and Shannon diversity of plants, while random effects include month, habitat types, recorder position (start and end transect), and transect code. We did not include humidity as a predictor due to its high value in collinearity with temperature. Among all variables, ACI does not result any significant p-value (Fig 6). ADI is correlated with temperature and Shannon diversity of plant. AEI is correlated with temperature and canopy cover. BI is correlated with temperature.

### Habitat features

In total, we documented 194 vascular plant species, comprising both woody trees and understory vegetation such as herbaceous plants, ferns, vines, shrubs, lianas, and grasses, with a total count of 2,845 individuals. Tree growth stages, ranging from seedling to mature trees, were observed across disturbed peat forests, less disturbed peat forests, and disturbed wet shrub, while only seedlings were noted in estate crop areas (S13 Fig). Understory vegetation was found in all habitat types. However, a notable number of understoreys were observed in non-forested areas (estate crops and disturbed wet shrub) with peatland-typical undergrowth species such as *Nephrolepis biserrata* (ferns, n = 140 in estate crop, n = 176 in wet shrub), *Pteridium esculentum* (ferns, n = 19 in estate crop, n = 132 in wet shrub), and *Stenochlaena palustris* (ferns, n = 55 in estate crop, n = 170 in wet shrub) becoming dominant species inhabiting non-forested areas. The undergrowth species were different in disturbed peat forests, where although *Nephrolepis biserrata* (n = 22) remains the most abundant species, others such as *Freycinetia angustifolia* (liana, n = 18), *Hanguana sp.* (herb, n = 15), and *Nepenthes ampullaria* (liana, n = 10) were also abundant. Understory species found in disturbed peat forests were observed in very low numbers in less disturbed peat forests.

A high number of individual and species counts from saplings were recorded in disturbed and less disturbed peat forests. In forested areas, we found a smaller number of understory individuals compared to what we found in estate crops and disturbed wet shrub areas. Consistent across all growth phases of trees, less disturbed peat forests have higher total individual and species counts compared to disturbed peat forests. However, compared to disturbed peat forests, the less disturbed peat forest has fewer total individual and species in the understory.

Forest areas also harbour more Critically Endangered plant species compared to non-forest areas such as *Gonystylus bancanus* and *Shorea platycarpa*, and Endangered species *Shorea teysmanniana*, as well as protected species, i.e., *Koompassia malaccensis* and *Nepenthes bicalcarata*, and endemic species. Between forested areas, less disturbed forest has more critically endangered and protected species than disturbed forest. However, disturbed peat forest also has more endemic plant species and Conservation Dependent species. The non-forested areas on the other hand, harbor fewer plant species and zero high-risk plant species, except for one Near Threatened species, i.e., *Aglaia rubiginosa*. Despite having lower plant diversity, non-forested area still provides great amount of ground cover.

The highest plant individual count was recorded in less disturbed peat forests, followed by disturbed peat forests, estate crops, and disturbed wet shrubs. Similarly, less disturbed peat forests exhibited the highest species count, with disturbed wet shrub recording the lowest. The species count was comparable between forested (disturbed and less disturbed peat forests) and non-forested (estate crop and disturbed wet shrub) areas. Forested areas had higher species count than non-forested area. However, the pattern did not apply to the plant individual counts, as disturbed peat forests, estate crop, and disturbed wet shrubs all had similar numbers.

**Fig 7:**
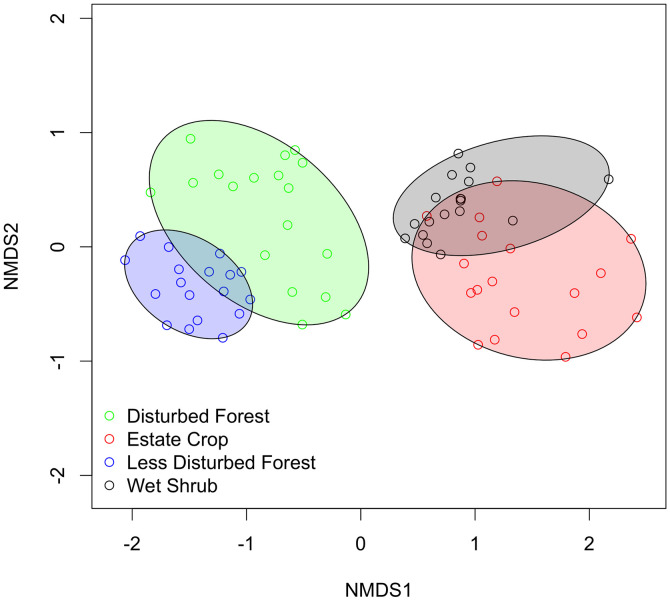
NMDS ordination plot showing plant species composition of four habitat types.

Dissimilarity between forested and non-forested areas was observed in plant species composition and thus reflected in Shannon diversity index. NMDS plot shows overlap between disturbed peat forests with less disturbed peat forest and estate crop with disturbed wet shrubs (Fig 7). The separation between forested or non-forested areas using NMDS indicate that there are still species we found only specific to a habitat types. As many as 65 species recorded were only found in less disturbed peat forests, 56 species in disturbed peat forests, 15 in estate crops, and 7 species only occur at disturbed wet shrub. Disturbed peat forests and less disturbed peat forests have higher plant diversity index compared to estate crop areas and disturbed wet shrub.

On average, tree height and diameter were higher in forested areas compared to disturbed wet shrub. However, within forested areas, trees with diameter wider than 45 cm and height above 30 m were only found in less disturbed peat forest. Canopy cover was present in all habitat types but very low number in disturbed wet shrub (mean = 1.97%). In estate crops, canopy cover was due to shade from oil palm stands. Estate crops had lower canopy cover than disturbed and less disturbed peat forest. All our survey areas in estate crop took place in palm oil plantations with height of each plant reach up to more than 2 m. Ground cover was observed across all habitat types. Mean percentage of canopy and ground cover was highest in less disturbed peat forest followed by disturbed peat forest. Among all habitats, less disturbed peat forest had the highest average of tree height, tree diameter, ground cover and canopy cover.

**Fig 8:**
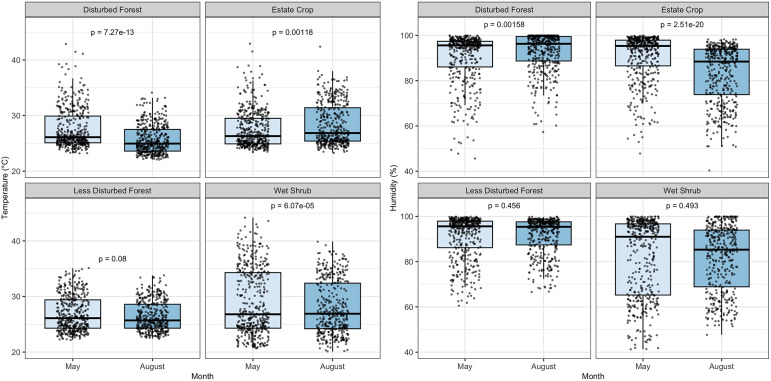
Boxplot of temperature and humidity for four habitat types. P-values for pairwise comparisons between months within each habitat were obtained from linear mixed-effects models with habitat, transect code, and transect position as nested random effects. Post-hoc tests were adjusted by Bonferroni correction.

Temperature and humidity were relatively stable across months in less disturbed forest (Fig 8). Temperature ranges from 20° to 44°C. The highest humidity was 99.99% while the lowest was in 40.3%. Temperature and humidity tend to have an inverse relationship. In both survey months, humidity was lower during dawn to morning time and peaked when approaching noon, usually around 10:00–15:00. Temperature declined after noon approaching nighttime and remained stable until dawn. On the contrary, lowest humidity was recorded during the period 10:00–15:00. Humidity increased until nighttime and remained stable but high until morning, comparable to the pattern of temperature. This pattern was consistent across all four habitat types. The median temperature in disturbed and less disturbed peat forests was relatively lower than in estate crops and disturbed wet shrubs. The median humidity was relatively higher in disturbed and less disturbed peat forest compared to estate crops and disturbed wet shrubs.

## Discussion

Within the rewetting sites, we found a clear pattern in wildlife attributes between forested and non-forested areas. Estate crop as a non-forested habitat is different from disturbed and less disturbed peat forest in terms of wildlife diversity (Shannon diversity index, Simpson diversity index, acoustic diversity index, bioacoustics index), wildlife richness (Menhinick’s Richness Index, Margalef’s Richness Index), wildlife evenness (Pielou evenness index and acoustic evenness index), and wildlife dominance (Berger Parker Dominance Index). Disturbed and less disturbed peat forest is found to be similar in all wildlife variables, except for the acoustic complexity index. Forested area has higher wildlife diversity, richness, and evenness, also lower dominance compared to non-forested area, particularly estate crop. This finding is also in line with similarity of plant species composition found within forested and non-forested area and differences between forested and non-forested area, with forested sites having higher plant diversity and richness.

Our study finds that habitat features affect wildlife in many ways. Wildlife richness was linked with habitat type, month, temperature, and humidity. Wildlife diversity was correlated with habitat type, temperature, understorey density, and plant diversity. Wildlife evenness was affected by habitat type, temperature, understorey density, and canopy cover. Wildlife dominance was related to habitat type, month, seedling density, and vegetation species richness.

Habitat types were likely to generate differences in habitat heterogeneity, with greater vegetation complexity affecting the findings because higher wildlife species richness indices were spotted in less disturbed and disturbed peat forests. Greater habitat complexity, including more diverse and varied vegetation, typically increases food and shelter availability for wildlife, fostering reproduction and survival, thus elevating species richness and total abundance [56–59]. Increased habitat complexity includes increasing number of canopy layers more likely to facilitate niche necessity between species. Regarding observation month, the dry season August survey identified higher species richness indices in all habitat types than the wet season survey in May and forested habitat still had greater richness than non-forested habitat. During the dry season, wildlife faces severe scarcity of food, water, and shelter, compelling species to relocate to habitats with more stable resources [60–63]. The increase in species richness indices from May to August across all rewetting sites demonstrates that peatlands play an important role in providing resources for wildlife, especially water, even at the peak of the dry season. Therefore, protecting peatlands from further disturbance and improve its quality is crucial.

Temperatures in forested habitats were lower, and humidity was higher, on average, than non-forested habitat. With higher means of humidity, make the habitat warm and humid at the same time, typical traits for tropical forest near equator such as Indonesia [64–66]. Such habitat characteristics influence wildlife fitness, and impacts can vary across species and populations. An increase in temperature outside of a species’ tolerance range may affect survival rates, leading to a decline in population [67]. Amphibians are greatly influenced by their environment, where shifts in temperature and moisture levels can impact survival, distribution, and population size [68,69]. Reptiles are temperature sensitive and increasing temperatures could affect their thermoregulation patterns and reproduction [70]. Additionally, temperature also impacts their population abundance, behaviour, and energetic costs of numerous bird and mammal species [67,71]. Higher wildlife richness, diversity, and evenness in forested habitat demonstrates that the habitat provides more ideal temperature and humidity for most of species than open habitat such as estate crop and disturbed wet shrub.

Understory vegetation holds significant importance, serving as both food for herbivores and shelter for ground-nesting birds and small mammals [72]. In addition, understory also offers additional niches and shelter, providing protection against heat and predation for herpetofauna [73–76]. Despite the vital role understory plays in wildlife diversity, it does not necessarily imply that habitats solely comprised of understory cover are most suitable for wildlife. Our results show that complex habitats with multiple vegetation layers may be more advantageous for some species. Understory becomes particularly crucial in plantations, which typically consist of homogeneous species. The presence of understory cover adds an additional layer to the habitat. Thus, when intensive plantation management removes the understory layer, often considered as weeds, it will reduce the occurrence of fauna in the habitat [77].

Dominance and evenness are two interconnected concepts. Evenness pertains to how similar the abundances of co-existing species are, while dominance of one or a few species is evident when there is unevenness in species abundances [78]. When one species dominates a habitat, there is low evenness, whereas the absence of dominant species suggests high evenness. The value of Pielou’s Evenness and Berger Parker Dominance Index illustrates that estate crop has lower evenness, thus lower diversity, because it is dominated by the Yellow-vented Bulbul (*Pycnonotus goiavier*). In May, we observed 55 individuals of this bird species, while in August, the count was 49 individuals. According to the GLM analysis, there is an inverse correlation between the Berger Parker Dominance Index and the diversity of vegetation species. The homogeneous palm oil plantation may only provide resources for small range species. The Yellow-vented bulbul as a generalist species demonstrates adaptability to various habitats including mangroves, secondary forests, rural, and urban areas [79]. The bulbul appears to have effectively adapted to the resources provided in palm oil plantations by utilizing insects and palm oil fruit remnants as food sources [80]. Consequently, it emerged as the most sighted species within the oil palm plantation. In other habitat types, we also encountered the Yellow-vented Bulbul, but the abundance was not as massive as we found in the palm oil plantation. By reviewing the pattern of dominance and evenness indices, we can infer that a complex habitat tends to promote greater evenness.

Passive acoustic monitoring led to detection of finer scale differences in disturbed wet shrub when compared to results from the point-count and line transect surveys. Acoustics indices (particularly ADI, AEI, and BI) differentiated disturbed wet shrub and forested areas, and similarity with estate crop. Since frequency range used to calculate acoustics indices can also detect insect vocalization, it is possible that more insect species were detected, thus increasing the diversity [81–83]. Passive acoustic monitoring resulted in the same environmental and habitat features as explanatory variables, that more likely to directly affect taxa detection rather than to the actual number of acoustic indices [84].

The differing methodologies employed in our study complement each other, providing a comprehensive understanding of the habitats under investigation. Direct sighting of wildlife provides a baseline to conduct macro taxa detection during acoustic analysis. In addition, the continuous audio recording allows us to capture any species present which we did not encounter in person due to limitations in conducting continuous human observation. The calculation of acoustic indices also granted a direct approach to acoustic diversity as complementary approach to assessing to biological diversity, although interpretation requires careful considerations. We did not use camera traps in this survey due to security concerns, the lower probability of immediate results within our study timeframe, and the shorter detection range. If possible, the further study can apply camera trap to complete human observation and acoustic monitoring because it is useful in capturing non-vocal animal, particularly mammals [85].

During our surveys, the only restoration effort observed for degraded peatlands was canal blocking. This involved constructing dams to retain water and reduce rapid drainage during the dry season, ultimately aiming to raise the water table and restore the hydrological function of the peatland after extensive canal construction had drained it [86]. However, a study on peat rewetting in a boreal landscape reported that while improvements in water storage and runoff dynamics were observed, full hydrological recovery was not evident within the first three years [87]. Canal blocking has also been successful in preventing fire and haze in several locations [88,89], but our sites have yet to experience the benefit. This was highlighted by a fire that occurred at our sites and their surroundings on August 23, 2024, when this study was conducted, posing a threat to biodiversity. Furthermore, when considering the direct impact of canal blocking on biodiversity in our sites, there is insufficient evidence to suggest its effect on wildlife diversity in all habitats. The higher wildlife diversity observed in forested areas appears to be influenced by their complex vegetation structure, which creates an ideal microclimate and resource availability for the wildlife. In contrast, non-forested habitats have lower wildlife diversity due to their open conditions and limited vegetation structure, which do not provide a suitable environment for a diverse range of species. Nevertheless, the fact that vegetation, regardless of its woody trait, can still be found in estate crop sites and disturbed wet shrub underscores the indication that they might still be suitable habitats, given improved management for wildlife. Non-forested areas still provide ground cover and canopy shades for inhabiting wildlife species, thus, may likely to contribute to biodiversity in degraded peatlands of West Kalimantan, although may not as significantly as forested habitats. Similar results have also been reported, indicating that canal blocking contributes to habitat improvement. After 7.5 years of monitoring, a constructed dam in a peat swamp forest canal resulted in halved rates of peat surface subsidence and the spontaneous regrowth of 57 native tree species [90]. These findings suggest the importance of long-term observation to fully understand the mechanisms by which canal blocking influences wildlife dynamics. It is also essential to statistically account for other interconnected factors, such as anthropogenic and climatic variables, as these are linked to wildlife community, species distribution and habitat use [91–93].

To enhance wildlife diversity in our study site, canal blocking alone is insufficient for rehabilitating degraded peatlands. It represents just the initial step in an overall strategy aimed at reducing fire risk and supporting revegetation efforts [94,95]. We propose aligning biodiversity conservation efforts at the site with the natural climate solutions (NCS) hierarchy, which is part of nature-based solutions (NbS) and aims to reduce GHG emissions or enhance carbon sequestration while protecting biodiversity and human well-being [96]. NCS hierarchy begins with land protection and progresses to improve management and restoration [97]. In the NCS hierarchy, protection is the priority action, offering a large area of mitigation in a short time and being highly cost-effective with numerous co-benefits. The second action in the hierarchy is improved management, which provides a lower-cost mitigation potential than restoration and requires minimal or no changes in land use. Restoration is third in the hierarchy because it is less cost-effective and requires land-use changes. Restoration NCS aim to rehabilitate or reestablish forest, wetland, and grassland ecosystems in areas where these habitats once naturally existed. This strategy is necessary for areas experiencing severe loss and degradation. Although the NCS hierarchy outlines a defined order, in practice, protection, improved management, and restoration can work together to achieve optimal results. Understanding the characteristics and needs of each habitat is crucial for choosing the appropriate NCS hierarchy strategy. The NCS hierarchy outlines a defined order, in practice, protection, improved management, and restoration can work together to achieve optimal results. Understanding the characteristics and needs of each habitat is crucial for choosing the appropriate NCS hierarchy strategy.

Our survey in the less disturbed peat forest within the Kubu Raya Landscape indicates that the forest has not yet reached climax in its ecological development and is still in the process of succession, dominated by trees with diameters of up to 20 meters. The peat forest that is still regenerating had canopy heights of 12–18 m while the canopy of mature forest can reach up to more than 45 m [98]. Since the less disturbed peat forest in the Kubu Raya Landscape has the potential to regenerate naturally, the primary management priority is to protect the area from further disturbance. This practice has indeed been applied in the area for approximately 10 years. In the past, the forest was a target for illegal logging, but after the area was designated as protected forest by the Permata Village Government, people rarely visited, and extractive activities fully ceased. The protection regulations have helped the forest recover from past disturbances and offer an ideal habitat for wildlife. The condition of the less disturbed peat forests in Kubu Raya, which have been locally protected for the past 10 years, demonstrates that if protection is applied by halting illegal logging, the forests can grow, support higher plant diversity, and serve as habitats for endangered plant species. [99].

The disturbed peat forest in the Mempawah Landscape exhibits a similar stage of ecological succession to the less disturbed forest in Kubu Raya, as both are still undergoing natural succession. However, ongoing disturbances continue to impact the forest in the Mempawah area. The most recorded undergrowth vegetation we found in disturbed peat forests of Mempawah is alike to that found colonized disturbed wet shrubs and estate crops. These species are typical pioneer, wind-dispersed fern species [100]. These are not usually found in mature peat forest but become abundant after disturbances such as land clearing and burning [100,101]. However, the presence of other abundant understory species (herb and woody lianas), trees, and canals only at the edge of the forest, suggests that disturbances have occurred some time ago and there have been no recent massive disturbances in disturbed peat forests [102]. These indications were confirmed during conversations with local people during our surveys. They stated that the forest is now under the management of the local forestry agency, with the primary concerns being forest fires, illegal wildlife hunting, and selective logging of certain tree species that have reached a certain age (usually the sapling stage) or a diameter of less than 10 cm. During our surveys, we neither observed nor heard of any actions regarding the replanting of vegetation due to legal restrictions imposed on disturbed forest areas, suggesting that any vegetation growth occurs naturally.

Protection is the NCS strategies also needed by the disturbed peat forest, as these forests have the potential to regenerate naturally and only require support to boost recovery. Research has shown that a peat forest in Central Kalimantan that experienced a single burning event in 1997 demonstrated similar Shannon diversity and plant species counts to unburned forests after 13 years [99]. Similarly, research from Central Kalimantan also demonstrates that peat forests subjected to selective logging in the past have a strong natural regeneration potential, shown by their high resemblance to relatively undisturbed forests in terms of tree density, species richness, and a similar understory vegetation community [103]. Additionally, if illegal hunting is curtailed, the forest could benefit from the presence of mammals and birds, which would serve as seed dispersers and pollinators to aid in the regeneration process [104]. Despite instances of illegal activity such as burn-clearing, logging, and hunting, disturbed peat forests in Mempawah Landscape still hold promise as potential habitats, and, if left to regenerate naturally with interventions focused on illegal activities protection, they can progress into more mature forests with high biodiversity [105].

In estate crops where peatland has already been converted to oil palm plantations, the NCS hierarchy to support biodiversity conservation and increase carbon sequestration includes protection and improved management. Since the vegetation complexity of estate crops cannot provide suitable habitats for a wide range of wildlife species, protection from hunting—which still occurs in these areas—is crucial to maintain existing biodiversity. Hunting, especially targeting rare and endangered species, needs to be minimized as much as possible, as some wildlife have large area coverage, which is a consequence of establishing a plantation in a previously forested area [106]. Moreover, according to our results, one factor that supports estate crops as wildlife habitats is the presence of understory vegetation. The plantation management protocols in oil palm plantation, such as undergrowth clearing for fruit harvesting, road development, pest and weed management, or tillage, can potentially eliminate understory coverage, impacting the biodiversity of the estate crop [107–109]. Maintaining understory coverage in estate crops can be considered an improved management practice. The presence of understory plants does not compete with oil palms; instead, it provides multiple benefits, such as reducing CO₂ emissions, increasing carbon stocks, enhancing biodiversity, improving water and soil nutrient balances, and reducing erosion [110,111].

Furthermore, replacing the homogeneous structure with heterogeneity in oil palm plantations can enhance habitat quality to sustain forest-like areas [112]. Oil palm plantations in peatland areas are well-known actors in the cascading effects of habitat quality degradation, one of which is biodiversity loss. In addition, plantations can harbour species, usually common species, which are sometimes considered pests and potentially invasive and in long-term could increase the wild crop-raiding population thus affecting the forest [113]. The estate is also not being used by forest-specific species as habitat, especially those that are peat forest specialists [109]. Adopting the jangka benah strategy—a solution to address unauthorized oil palm plantations in forest areas by implementing oil palm agroforestry—could be a suitable practice for estate crops to diversify vegetation [114]. Rather than expanding land area, intensification within existing areas will likely increase yield and optimize biodiversity conservation [115]. Participation in oil palm certifications (roundtable on sustainable palm oil (RSPO) or Indonesian sustainable palm oil (ISPO) by companies or smallholders is another form of improved management that can help prevent biodiversity loss, with recent reports indicating positive impacts of these certifications on biodiversity [116].

The disturbed wet shrub areas in the Mempawah Landscape, which are dominated by open areas, require more intensive recovery actions. For disturbed wet shrublands already used for agriculture, improved management practices need to be applied. An agroforestry system that enriches the land with woody plants offers an alternative for farmers to increase yield potential while contributing to biodiversity conservation and climate change mitigation. In addition to agricultural use, some disturbed wet shrub areas are left open. For these areas, protection should be prioritized, as fire regimes pose one of the greatest threats to dry peatlands, especially since canals have disrupted their hydrological systems [15,117]. In disturbed wet shrub areas that experience recurring fires, the third level of NCS, which is restoration, is the only way to recover the area.

Repeated burning proved to prevent regeneration [101]. Studies show that a single forest fire can transform an initially forested area into an open area, but regeneration can occur relatively quickly due to the fire’s facilitation of seed sprouting for some species [99,100]. However, recurring fires can deplete soil nutrients, reduce soil fertility, diminish canopy, and ground cover, eliminate vegetation roots, and ultimately halt the process of plant regrowth and seed sprouting, resetting the succession to level zero [101,118,119] This chain reaction can significantly decrease biodiversity levels and increase the dominance of specific plants, such as herbaceous ferns, in an area. This condition aligns with the observation results drawn from disturbed wet shrub [120]. With the worsening effects of rising global temperatures and El Niño periods, there is a drive to improve attitudes and strengthen restoration projects like never before [15,121,122]. Repeated fires are one of the factors halting open peatland revegetation. It is crucial to consider this when conducting restoration in vast peat wet shrublands.

With environmental deterioration from many aspects, restoration projects need to be implemented hand in hand [105]. On the rewetting sites, vegetation planting needs to incorporate local communities into action. This makes sense not only in terms of effective funding but also by providing benefits for the community, such as allowing them to select local trees that potentially increase their well-being through non-timber forest products or adopting responsible sustainable logging practices [105,123–125]. Involving local champions in building local nurseries for peatland revegetation and decision-making in peatland restoration projects can also increase the chance of disturbed wet shrub recovery. After all, the presence of tree vegetation in the area can reduce the severity level of fire impacts, thus preventing the global hazard caused by massive landscape fire [118,126].

## Supporting information

S1 Fig Boxplot showing acoustic indices from four habitats. (TIF)

S2 Table Acoustic index statistics across habitat types. (XLSX)

S3 Table Acoustic index statistics across habitat types and months. (XLSX)

S4 Table Habitat statistics. (XLSX)

S5 Table Temperature and humidity statistics across habitat types. (XLSX)

S6 Table Generalized Linear Model (GLM) of wildlife survey. (DOCX)

S7 Table Result of analysis of deviance table (Type II tests) for the GLM model for wildlife survey. (DOCX)

S8 Table Post hoc test using Tukey HSD for wildlife survey. (DOCX)

S9 Table Analysis of deviance table (Table II Wald chi-square tests). (DOCX)

S10 Table Pairwise differences of acoustic indices in each habitat type. (DOCX)

S11 Table Summary result of Linear Mixed Model from acoustic indices. (DOCX)

S12 Table Number of individual and species for every growth stage in each habitat. (DOCX)

S13 Fig Boxplot showing vegetation features from four habitats. (TIF)

S14 File Inclusivity in global research questionnaire. (DOCX)
