# Supplementary figures and images for: Effect of canal blocking on biodiversity of degraded peatlands: Insight from West Kalimantan
Source: PLoS One. 2025 Oct 8;20(10):e0334014. doi: 10.1371/journal.pone.0334014 (PMC12507311; doi:10.1371/journal.pone.0334014)

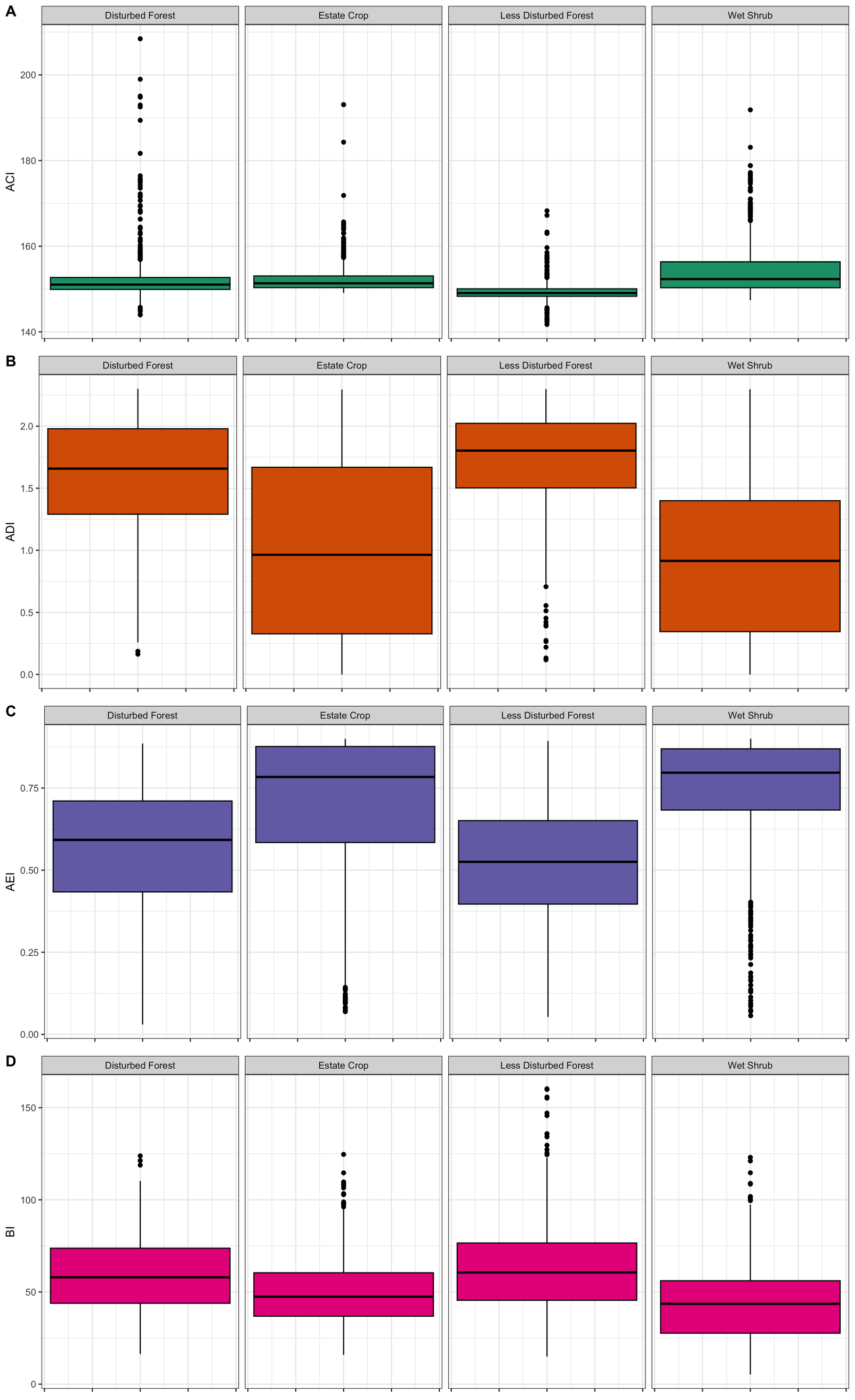

Supplement: S1 Fig — (TIF) [file pone.0334014.s001.tif]

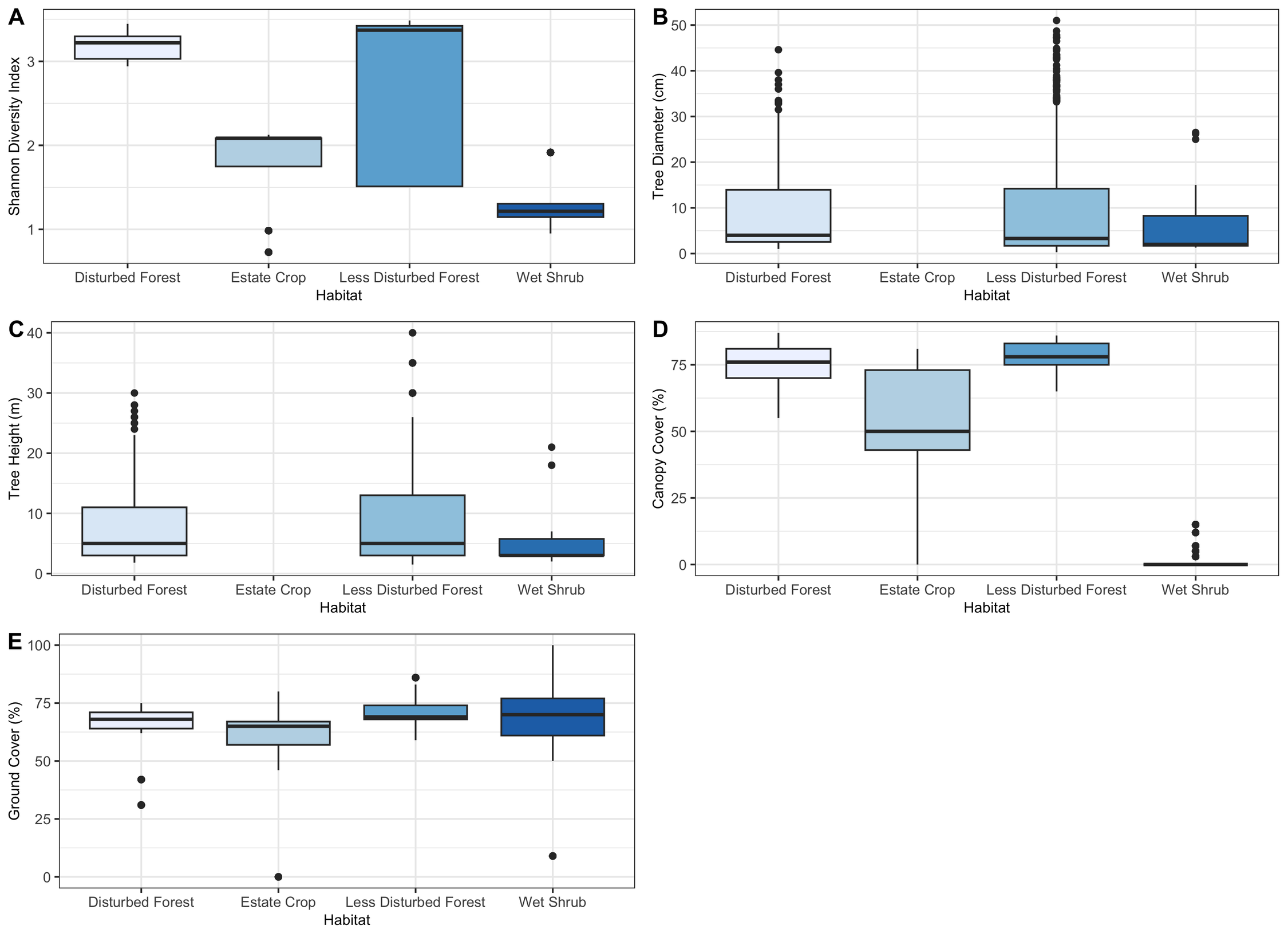

Supplement: S13 Fig — (TIF) [file pone.0334014.s013.tif]
