# Supplementary material for: Effect of canal blocking on biodiversity of degraded peatlands: Insight from West Kalimantan
Source: PLoS One. 2025 Oct 8;20(10):e0334014. doi: 10.1371/journal.pone.0334014 (PMC12507311; doi:10.1371/journal.pone.0334014)
Supplement: S6 Table — (DOCX) [file pone.0334014.s006.docx]

S6 Table. Generalized Linear Model (GLM) of wildlife survey

| **Responses** | **Predictors** | **Estimate** | **Std.Error** | **t-Value** | **Pr(>\|t\|)** | **Signif.Codes** |
| --- | --- | --- | --- | --- | --- | --- |
| Number of Wildlife Individual | (Intercept) | 6.05E+00 | 1.68E+01 | 0.361 | 0.72199 |  |
|  | canopyCover | 1.45E-01 | 8.82E-02 | 1.649 | 0.11559 |  |
|  | groundCover | 3.36E-01 | 1.98E-01 | 1.694 | 0.10666 |  |
|  | understoreyDensity | 6.42E-05 | 2.02E-05 | 3.175 | 0.00499 | ** |
|  | monthMay | 1.10E+01 | 3.17E+00 | 3.466 | 0.00259 | ** |
| Species Richness | (Intercept) | -86.8821 | 41.5846 | -2.089 | 0.05115 | . |
|  | habitatDisturbedForest | 6.5823 | 2.1346 | 3.084 | 0.00641 | ** |
|  | habitatLess DisturbedForest | 4.8925 | 1.8 | 2.718 | 0.0141 | * |
|  | habitatWetShrub | 2.6223 | 1.6646 | 1.575 | 0.13258 |  |
|  | temperature | 2.5797 | 1.033 | 2.497 | 0.02244 | * |
|  | humidity | 0.3489 | 0.2077 | 1.679 | 0.11035 |  |
| Shannon Diversity Index | (Intercept) | -2.76E+00 | 2.52E+00 | -1.095 | 0.28888 |  |
|  | habitatDisturbedForest | 7.88E-01 | 2.16E-01 | 3.642 | 0.00202 | ** |
|  | habitatLess DisturbedForest | 7.47E-01 | 2.20E-01 | 3.395 | 0.00344 | ** |
|  | habitatWetShrub | 2.45E-01 | 1.01E-01 | 2.424 | 0.02677 | * |
|  | temperature | 1.20E-01 | 6.28E-02 | 1.907 | 0.07363 | . |
|  | humidity | 1.63E-02 | 1.28E-02 | 1.276 | 0.21897 |  |
|  | understoreyDensity | 9.63E-07 | 6.41E-07 | 1.504 | 0.15105 |  |
| Simpson Diversity Index | (Intercept) | 8.25E-01 | 2.15E-02 | 38.418 | <2.00E-16 | *** |
|  | habitatDisturbedForest | 1.05E-01 | 1.98E-02 | 5.304 | 4.05E-05 | *** |
|  | habitatLess DisturbedForest | 1.11E-01 | 2.21E-02 | 5.015 | 7.68E-05 | *** |
|  | habitatWetShrub | 4.14E-02 | 1.06E-02 | 3.919 | 0.00092 | *** |
|  | understoreyDensity | 1.38E-07 | 7.16E-08 | 1.922 | 0.06967 | . |
| Pielou's Evenness Index | (Intercept) | 7.81E-01 | 2.66E-02 | 29.36 | <2.00E-16 | *** |
|  | habitatDisturbedForest | 1.29E-01 | 2.46E-02 | 5.242 | 4.64E-05 | *** |
|  | habitatLess DisturbedForest | 1.35E-01 | 2.74E-02 | 4.915 | 9.61E-05 | *** |
|  | habitatWetShrub | 3.86E-02 | 1.31E-02 | 2.951 | 0.00821 | ** |
|  | understoreyDensity | 1.81E-07 | 8.87E-08 | 2.04 | 0.0555 | . |
| Berger Parker Dominance Index | (Intercept) | 3.95E-01 | 2.85E-02 | 13.866 | 2.47E-10 | *** |
|  | habitatDisturbedForest | -3.41E-02 | 7.92E-02 | -0.431 | 0.67221 |  |
|  | habitatLess DisturbedForest | -2.34E-02 | 1.28E-01 | -0.184 | 0.85664 |  |
|  | habitatWetShrub | -1.19E-01 | 2.57E-02 | -4.627 | 0.00028 | *** |
|  | saplingDensity | 1.35E-05 | 9.63E-06 | 1.397 | 0.1815 |  |
|  | seedlingDensity | -1.49E-06 | 6.76E-07 | -2.197 | 0.04309 | * |
|  | numberVegetationSpecies | -4.37E-03 | 1.49E-03 | -2.939 | 0.00962 | ** |
|  | monthMay | -2.85E-02 | 1.71E-02 | -1.668 | 0.11473 |  |
| Menhinick's Richness Index | (Intercept) | -15.04326 | 6.17112 | -2.438 | 0.02606 | * |
|  | habitatDisturbedForest | 1.25763 | 0.21518 | 5.844 | 1.95E-05 | *** |
|  | habitatLess DisturbedForest | 0.97052 | 0.16975 | 5.717 | 2.52E-05 | *** |
|  | habitatWetShrub | 0.44723 | 0.16184 | 2.763 | 0.01329 | * |
|  | temperature | 0.44758 | 0.14746 | 3.035 | 0.00747 | ** |
|  | humidity | 0.05523 | 0.02797 | 1.974 | 0.06481 | . |
|  | monthMay | -0.39688 | 0.15845 | -2.505 | 0.02273 | * |
| Margalef's Richness Index | (Intercept) | -30.41885 | 13.5585 | -2.244 | 0.03847 | * |
|  | habitatDisturbedForest | 2.06369 | 0.47277 | 4.365 | 0.00042 | *** |
|  | habitatLess DisturbedForest | 1.52833 | 0.37295 | 4.098 | 0.00075 | *** |
|  | habitatWetShrub | 0.7922 | 0.35558 | 2.228 | 0.03968 | * |
|  | temperature | 0.86061 | 0.32399 | 2.656 | 0.01662 | * |
|  | humidity | 0.11771 | 0.06146 | 1.915 | 0.07242 | . |
|  | monthMay | -0.52882 | 0.34814 | -1.519 | 0.14714 |  |
