# Supplementary material for: Effect of canal blocking on biodiversity of degraded peatlands: Insight from West Kalimantan
Source: PLoS One. 2025 Oct 8;20(10):e0334014. doi: 10.1371/journal.pone.0334014 (PMC12507311; doi:10.1371/journal.pone.0334014)
Supplement: S7 Table — (DOCX) [file pone.0334014.s007.docx]

S7 Table. Result of Analysis of Deviance Table (Type II tests) for the GLM model for wildlife survey

| Responses | Predictors | LR Chisq | Df | Pr(>Chisq) | Signif. codes |
| --- | --- | --- | --- | --- | --- |
| Number of Wildlife Individual | canopyCover | 2.7191 | 1 | 0.0991515 | . |
|  | groundCover | 2.8686 | 1 | 0.0903248 | . |
|  | understoreyDensity | 10.079 | 1 | 0.0014997 | ** |
|  | month | 12.0157 | 1 | 0.0005275 | *** |
| Species Richness | habitat | 11.1857 | 3 | 0.01076 | * |
|  | temperature | 6.2363 | 1 | 0.01252 | * |
|  | humidity | 2.8204 | 1 | 0.09307 | . |
| Shannon Diversity Index | habitat | 18.6865 | 3 | 0.0003174 | *** |
|  | temperature | 3.6348 | 1 | 0.0565832 | . |
|  | humidity | 1.6293 | 1 | 0.2018026 |  |
|  | understoreyDensity | 2.2607 | 1 | 0.1326971 |  |
| Simpson Diversity Index | habitat | 34.767 | 3 | 1.36E-07 | *** |
|  | understoreyDensity | 3.696 | 1 | 0.05455 | . |
| Pielou's Evenness Index | habitat | 30.102 | 3 | 1.31E-06 | *** |
|  | understoreyDensity | 4.1616 | 1 | 0.04135 | * |
| Berger Parker Dominance Index | habitat | 21.7411 | 3 | 7.38E-05 | *** |
|  | saplingDensity | 1.9515 | 1 | 0.16243 |  |
|  | seedlingDensity | 4.8272 | 1 | 0.02801 | * |
|  | numberVegetationSpecies | 8.6392 | 1 | 0.00329 | ** |
|  | month | 2.7828 | 1 | 0.09528 | . |
| Menhinick's Richness Index | habitat | 40.827 | 3 | 7.12E-09 | *** |
|  | temperature | 9.213 | 1 | 0.002403 | ** |
|  | humidity | 3.898 | 1 | 0.048337 | * |
|  | month | 6.273 | 1 | 0.012257 | * |
| Margalef's Richness Index | habitat | 22.3404 | 3 | 5.54E-05 | *** |
|  | temperature | 7.0561 | 1 | 0.007899 | ** |
|  | humidity | 3.6687 | 1 | 0.055443 | . |
|  | month | 2.3074 | 1 | 0.128761 |  |
