# Supplementary material for: Effect of canal blocking on biodiversity of degraded peatlands: Insight from West Kalimantan
Source: PLoS One. 2025 Oct 8;20(10):e0334014. doi: 10.1371/journal.pone.0334014 (PMC12507311; doi:10.1371/journal.pone.0334014)
Supplement: S8 Table — (DOCX) [file pone.0334014.s008.docx]

S8 Table. Post Hoc Test Using Tukey HSD for wildlife survey

| **Habitat** | **diff** | **lwr** | **upr** | **p adj** |
| --- | --- | --- | --- | --- |
| **Wildlife Individual Count** |  |  |  |  |
| Disturbed Forest-Estate Crop | -9.6666667 | -26.8015 | 7.468168 | 0.412286 |
| Less Disturbed Forest-Estate Crop | -9.3333333 | -26.46817 | 7.801501 | 0.4421834 |
| Wet Shrub-Estate Crop | -4 | -21.13483 | 13.134834 | 0.9131181 |
| Less Disturbed Forest-Disturbed Forest | 0.3333333 | -16.8015 | 17.468168 | 0.99994 |
| Wet Shrub-Disturbed Forest | 5.6666667 | -11.46817 | 22.801501 | 0.7915732 |
| Wet Shrub-Less Disturbed Forest | 5.3333333 | -11.8015 | 22.468168 | 0.8195383 |
| **Wildlife Species Richness** |  |  |  |  |
| Disturbed Forest-Estate Crop | 4.0000000 | -0.6180090 | 8.6180090 | 0.1045052 |
| Less Disturbed Forest-Estate Crop | 3.6666667 | -0.9513424 | 8.2846760 | 0.1512970 |
| Wet Shrub-Estate Crop | 2.0000000 | -2.6180090 | 6.6180090 | 0.6265453 |
| Less Disturbed Forest-Disturbed Forest | -0.3333333 | -4.9513424 | 4.2846760 | 0.9969822 |
| Wet Shrub-Disturbed Forest | -2.0000000 | -6.6180090 | 2.6180090 | 0.6265453 |
| Wet Shrub-Less Disturbed Forest | -1.6666667 | -6.2846757 | 2.9513420 | 0.7454277 |
| **Shannon Diversity Index** |  |  |  |  |
| Disturbed Forest-Estate Crop | 0.44321605 | 0.17235153 | 0.71408057 | 0.0009635 |
| Less Disturbed Forest-Estate Crop | 0.42943161 | 0.15856709 | 0.70029613 | 0.0013337 |
| Wet Shrub-Estate Crop | 0.21064641 | -0.06021811 | 0.48151093 | 0.1639729 |
| Less Disturbed Forest-Disturbed Forest | -0.01378444 | -0.28464896 | 0.25708008 | 0.998933 |
| Wet Shrub-Disturbed Forest | -0.23256965 | -0.50343417 | 0.03829487 | 0.1087409 |
| Wet Shrub-Less Disturbed Forest | -0.21878521 | -0.48964973 | 0.05207931 | 0.1412525 |
| **Pielou's Evenness Index** |  |  |  |  |
| Disturbed Forest-Estate Crop | 0.086373189 | 0.046993547 | 0.12575283 | 0.0000296 |
| Less Disturbed Forest-Estate Crop | 0.085626472 | 0.046246829 | 0.12500612 | 0.0000333 |
| Wet Shrub-Estate Crop | 0.037692832 | -0.00168681 | 0.07707248 | 0.0637094 |
| Less Disturbed Forest-Disturbed Forest | -0.000746718 | -0.04012636 | 0.03863293 | 0.9999444 |
| Wet Shrub-Disturbed Forest | -0.048680358 | -0.08806 | -0.00930072 | 0.0121645 |
| Wet Shrub-Less Disturbed Forest | -0.04793364 | -0.08731328 | -0.008554 | 0.0136767 |
| **Berger Perker Dominance Index** |  |  |  |  |
| Disturbed Forest-Estate Crop | -0.13807514 | -0.21983383 | -0.05631645 | 0.0006892 |
| Less Disturbed Forest-Estate Crop | -0.15063734 | -0.23239603 | -0.06887865 | 0.00026 |
| Wet Shrub-Estate Crop | -0.09155399 | -0.17331268 | -0.0097953 | 0.0247508 |
| Less Disturbed Forest-Disturbed Forest | -0.0125622 | -0.09432089 | 0.06919649 | 0.9726195 |
| Wet Shrub-Disturbed Forest | 0.04652115 | -0.03523754 | 0.12827984 | 0.4049847 |
| Wet Shrub-Less Disturbed Forest | 0.05908335 | -0.02267534 | 0.14084204 | 0.213077 |
| **Menhinick's Richness Index** |  |  |  |  |
| Disturbed Forest-Estate Crop | 0.78030063 | 0.3289503 | 1.23165097 | 0.0005342 |
| Less Disturbed Forest-Estate Crop | 0.73366057 | 0.28231024 | 1.1850109 | 0.0010325 |
| Wet Shrub-Estate Crop | 0.36116414 | -0.09018619 | 0.81251447 | 0.1466984 |
| Less Disturbed Forest-Disturbed Forest | -0.04664007 | -0.4979904 | 0.40471027 | 0.9913063 |
| Wet Shrub-Disturbed Forest | -0.4191365 | -0.87048683 | 0.03221384 | 0.0746359 |
| Wet Shrub-Less Disturbed Forest | -0.37249643 | -0.82384676 | 0.0788539 | 0.1292178 |
| **Margalef's Richness Index** |  |  |  |  |
| Disturbed Forest-Estate Crop | 1.20950823 | 0.2624907 | 2.1565257 | 0.0094275 |
| Less Disturbed Forest-Estate Crop | 1.12542245 | 0.178405 | 2.07244 | 0.0163281 |
| Wet Shrub-Estate Crop | 0.57912052 | -0.367897 | 1.526138 | 0.3439906 |
| Less Disturbed Forest-Disturbed Forest | -0.08408577 | -1.0311033 | 0.8629317 | 0.994433 |
| Wet Shrub-Disturbed Forest | -0.63038771 | -1.5774052 | 0.3166298 | 0.2748571 |
| Wet Shrub-Less Disturbed Forest | -0.54630193 | -1.4933194 | 0.4007156 | 0.3932989 |
| **Temperature** |  |  |  |  |
| Disturbed Forest-Estate Crop | -1.7478781 | -2.8265786 | -0.66917761 | 0.0010668 |
| Less Disturbed Forest-Estate Crop | -1.0916673 | -2.1703677 | -0.01296677 | 0.0466689 |
| Wet Shrub-Estate Crop | 0.3174077 | -0.7612928 | 1.39610819 | 0.8426455 |
| Less Disturbed Forest-Disturbed Forest | 0.6562108 | -0.4224896 | 1.73491131 | 0.3483659 |
| Wet Shrub-Disturbed Forest | 2.0652858 | 0.9865853 | 3.14398627 | 0.0001652 |
| Wet Shrub-Less Disturbed Forest | 1.409075 | 0.3303745 | 2.48777544 | 0.0078584 |
| **Humidity** |  |  |  |  |
| Disturbed Forest-Estate Crop | 5.5225405 | 0.1585014 | 10.88658 | 0.0421758 |
| Less Disturbed Forest-Estate Crop | 4.5585928 | -0.8054463 | 9.922632 | 0.1138456 |
| Wet Shrub-Estate Crop | -4.1308152 | -9.4948543 | 1.233224 | 0.1701541 |
| Less Disturbed Forest-Disturbed Forest | -0.9639477 | -6.3279869 | 4.400091 | 0.957424 |
| Wet Shrub-Disturbed Forest | -9.6533557 | -15.0173948 | -4.289317 | 0.0003408 |
| Wet Shrub-Less Disturbed Forest | -8.689408 | -14.0534471 | -3.325369 | 0.0010697 |
| **Ground Cover** |  |  |  |  |
| Disturbed Forest-Estate Crop | 5.555556 | -18.2606 | 29.37171 | 0.8754829 |
| Less Disturbed Forest-Estate Crop | 11.5 | -12.31615 | 35.31615 | 0.4564706 |
| Wet Shrub-Estate Crop | 8.444444 | -15.37171 | 32.2606 | 0.6797514 |
| Less Disturbed Forest-Disturbed Forest | 5.944444 | -17.87171 | 29.7606 | 0.8529561 |
| Wet Shrub-Disturbed Forest | 2.888889 | -20.92726 | 26.70504 | 0.9787344 |
| Wet Shrub-Less Disturbed Forest | -3.055556 | -26.87171 | 20.7606 | 0.9750607 |
