# Supplementary material for: Effect of canal blocking on biodiversity of degraded peatlands: Insight from West Kalimantan
Source: PLoS One. 2025 Oct 8;20(10):e0334014. doi: 10.1371/journal.pone.0334014 (PMC12507311; doi:10.1371/journal.pone.0334014)
Supplement: S9 Table — (DOCX) [file pone.0334014.s009.docx]

S9 Table. Analysis of Deviance Table (Type II Wald chisquare tests)

| **Acoustic Index** | **Chisq** | **Df** | **Pr(>Chisq)** |
| --- | --- | --- | --- |
| **ACI** | 33.241 | 3 | 2.87E-07 |
| **ADI** | 91.618 | 3 | <2.2E-16 |
| **AEI** | 87.469 | 3 | <2.2E-16 |
| **BI** | 33.636 | 3 | 2.37E-07 |
