# Supplementary material for: Effect of canal blocking on biodiversity of degraded peatlands: Insight from West Kalimantan
Source: PLoS One. 2025 Oct 8;20(10):e0334014. doi: 10.1371/journal.pone.0334014 (PMC12507311; doi:10.1371/journal.pone.0334014)
Supplement: S10 Table — (DOCX) [file pone.0334014.s010.docx]

S10 Table. Pairwise differences of acoustic indices in each habitat type

| **Acoustic Index** | **Habitat** | **Estimate** | **Se** | **Df** | **Z.Ratio** | **P.Value** |
| --- | --- | --- | --- | --- | --- | --- |
| **ACI** | Estate Crop - Disturbed Forest | -0.0636 | 0.848 | Inf | -0.075 | 0.9998 |
|  | Estate Crop - Less Disturbed Forest | 2.9422 | 0.867 | Inf | 3.392 | 0.0039 |
|  | Estate Crop - Wet Shrub | -2.0292 | 0.848 | Inf | -2.392 | 0.0786 |
|  | Disturbed Forest - Less Disturbed Forest | 3.0058 | 0.867 | Inf | 3.465 | 0.003 |
|  | Disturbed Forest - Wet Shrub | -1.9656 | 0.848 | Inf | -2.317 | 0.0942 |
|  | Less Disturbed Forest - Wet Shrub | -4.9714 | 0.867 | Inf | -5.731 | <.0001 |
| **ADI** | Estate Crop - Disturbed Forest | -0.5801 | 0.0984 | Inf | -5.895 | <.0001 |
|  | Estate Crop - Less Disturbed Forest | -0.6552 | 0.1007 | Inf | -6.504 | <.0001 |
|  | Estate Crop - Wet Shrub | 0.1031 | 0.0984 | Inf | 1.048 | 0.7211 |
|  | Disturbed Forest - Less Disturbed Forest | -0.0752 | 0.1007 | Inf | -0.746 | 0.8784 |
|  | Disturbed Forest - Wet Shrub | 0.6832 | 0.0984 | Inf | 6.943 | <.0001 |
|  | Less Disturbed Forest - Wet Shrub | 0.7584 | 0.1007 | Inf | 7.528 | <.0001 |
| **AEI** | Estate Crop - Disturbed Forest | 0.1319 | 0.0265 | Inf | 4.975 | <.0001 |
|  | Estate Crop - Less Disturbed Forest | 0.1649 | 0.0271 | Inf | 6.075 | <.0001 |
|  | Estate Crop - Wet Shrub | -0.0493 | 0.0265 | Inf | -1.858 | 0.2462 |
|  | Disturbed Forest - Less Disturbed Forest | 0.033 | 0.0271 | Inf | 1.216 | 0.6169 |
|  | Disturbed Forest - Wet Shrub | -0.1811 | 0.0265 | Inf | -6.833 | <.0001 |
|  | Less Disturbed Forest - Wet Shrub | -0.2141 | 0.0271 | Inf | -7.89 | <.0001 |
| **BI** | Estate Crop - Disturbed Forest | -9.68 | 3.55 | Inf | -2.731 | 0.032 |
|  | Estate Crop - Less Disturbed Forest | -12.2 | 3.63 | Inf | -3.363 | 0.0043 |
|  | Estate Crop - Wet Shrub | 6.09 | 3.55 | Inf | 1.718 | 0.314 |
|  | Disturbed Forest - Less Disturbed Forest | -2.52 | 3.63 | Inf | -0.695 | 0.899 |
|  | Disturbed Forest - Wet Shrub | 15.77 | 3.55 | Inf | 4.45 | 0.0001 |
|  | Less Disturbed Forest - Wet Shrub | 18.3 | 3.63 | Inf | 5.042 | <.0001 |
