# Supplementary material for: Effect of canal blocking on biodiversity of degraded peatlands: Insight from West Kalimantan
Source: PLoS One. 2025 Oct 8;20(10):e0334014. doi: 10.1371/journal.pone.0334014 (PMC12507311; doi:10.1371/journal.pone.0334014)
Supplement: S11 Table — (DOCX) [file pone.0334014.s011.docx]

S1 Table. Summary result of Linear Mixed Model from acoustic indices

| **Acoustic Index** | **Fixed Effects** | **Estimate** | **Std. Error** | **df** | **t-value** | **Pr(>\|t\|)** | **Signif. Codes** |
| --- | --- | --- | --- | --- | --- | --- | --- |
| **ACI** | (Intercept) | 1.57E+02 | 3.13E+00 | 1.09E+01 | 50.221 | 3.15E-14 | *** |
|  | Temperature | 6.92E-03 | 1.89E-02 | 3.15E+03 | 0.366 | 0.714 |  |
|  | Canopy cover | -3.81E-02 | 2.45E-02 | 7.88E+00 | -1.555 | 0.159 |  |
|  | Ground cover | -2.90E-02 | 4.04E-02 | 1.90E+01 | -0.717 | 0.482 |  |
|  | Number of plant individuals | -5.30E-03 | 8.29E-03 | 1.60E+01 | -0.64 | 0.531 |  |
|  | Shannon diversity of plants | -2.65E-01 | 7.04E-01 | 1.43E+01 | -0.376 | 0.712 |  |
| **ADI** | (Intercept) | 1.44E+00 | 3.88E-01 | 7.95E+00 | 3.7 | 0.00611 | ** |
|  | Temperature | -3.73E-02 | 2.12E-03 | 3.31E+03 | -17.557 | < 2e-16 | *** |
|  | Canopy cover | 4.48E-03 | 2.42E-03 | 1.49E+01 | 1.854 | 0.08363 | . |
|  | Ground cover | 3.66E-03 | 4.53E-03 | 1.90E+01 | 0.807 | 0.42988 |  |
|  | Number of plant individuals | 6.26E-04 | 8.71E-04 | 1.82E+01 | 0.719 | 0.48154 |  |
|  | Shannon diversity of plants | 1.58E-01 | 7.32E-02 | 1.82E+01 | 2.158 | 0.0445 | * |
| **AEI** | (Intercept) | 5.16E-01 | 1.12E-01 | 4.19E+00 | 4.607 | 0.00894 | ** |
|  | Temperature | 1.23E-02 | 7.26E-04 | 3.32E+03 | 16.954 | < 2e-16 | *** |
|  | Canopy cover | -1.55E-03 | 5.92E-04 | 1.66E+01 | -2.609 | 0.01861 | * |
|  | Ground cover | -9.21E-04 | 1.06E-03 | 1.88E+01 | -0.867 | 0.39668 |  |
|  | Number of plant individuals | -1.31E-04 | 2.09E-04 | 2.02E+01 | -0.626 | 0.53861 |  |
|  | Shannon diversity of plants | -3.03E-02 | 1.75E-02 | 1.94E+01 | -1.733 | 0.09899 | . |
| **BI** | (Intercept) | 76.59603 | 10.84103 | 14.43728 | 7.065 | 4.75E-06 | *** |
|  | Temperature | -1.44153 | 0.08346 | 3314.46706 | -17.273 | < 2e-16 | *** |
|  | Canopy cover | 0.11761 | 0.07534 | 14.31258 | 1.561 | 0.14 |  |
|  | Ground cover | 0.08187 | 0.14382 | 18.68908 | 0.569 | 0.576 |  |
|  | Number of plant individuals | -0.02271 | 0.02748 | 17.39398 | -0.826 | 0.42 |  |
