# Supplementary material for: Effect of canal blocking on biodiversity of degraded peatlands: Insight from West Kalimantan
Source: PLoS One. 2025 Oct 8;20(10):e0334014. doi: 10.1371/journal.pone.0334014 (PMC12507311; doi:10.1371/journal.pone.0334014)
Supplement: S12 Table — (DOCX) [file pone.0334014.s012.docx]

S12 Table. Number of individual and species for every growth stage in each habitat

|  | **Total Individu** | **Species Richness** | **NT** | **VU** | **EN** | **CR** | **CD** | **Protected by law*** | **Borneo's endemic species** |
| --- | --- | --- | --- | --- | --- | --- | --- | --- | --- |
| **Estate Crop** | **564** | **25** | **1** |  |  |  |  |  |  |
| Understory | 506 | 25 |  |  |  |  |  |  |  |
| Seedling | 58 | 2 | 1 |  |  |  |  |  |  |
| **Disturbed Forest** | **566** | **102** | **12** | **5** | **1** | **1** | **2** | **1** | **7** |
| Understory | 84 | 11 | 1 |  |  |  |  |  |  |
| Seedling | 99 | 27 |  | 1 |  | 1 |  |  |  |
| Sapling | 256 | 69 | 6 | 5 |  |  | 1 |  | 5 |
| Pole | 61 | 34 | 3 | 1 | 1 |  | 1 |  | 2 |
| Tree | 66 | 32 | 4 |  |  |  | 2 | 1 | 4 |
| **Less Disturbed Forest** | **1176** | **106** | **5** | **4** | **1** | **2** | **1** | **2** | **2** |
| Understory | 17 | 7 |  | 1 |  |  |  | 1 | 1 |
| Seedling | 358 | 30 | 1 | 1 |  |  |  |  |  |
| Sapling | 557 | 79 | 5 | 3 |  | 2 | 1 | 1 | 1 |
| Pole | 80 | 39 | 1 | 2 | 1 |  |  |  |  |
| Tree | 164 | 51 | 3 | 1 | 1 | 2 |  |  | 1 |
| **Wet Shrub** | **539** | **20** | **1** |  |  |  |  |  |  |
| Understory | 497 | 9 |  |  |  |  |  |  |  |
| Seedling | 24 | 8 |  |  |  |  |  |  |  |
| Sapling | 14 | 5 |  |  |  |  |  |  |  |
| Pole | 1 | 1 |  |  |  |  |  |  |  |
| Tree | 3 | 2 | 1 |  |  |  |  |  |  |
| **Grand Total** | **2845** | **194** | **15** | **7** | **1** | **2** | **2** | **2** | **9** |

NT: Near Threatened, VU: Vulnerable, EN: Endangered, CR: Critically Endangered, CD: Conservation Dependent. Number presented in IUCN conservation status, protected species, and endemic species showed species count.

*P106: Minister of Environment and Forestry Regulation of The Republic of Indonesia Number P.106 / MENLHK / SETJEN / KUM.1 / 12/2018 on Second Amendment to The Minister of Environment and Forestry Regulation Number P.20 / MENLHK / SETJEN / KUM.1 / 6/2018 Regarding Protected Plant and Animal Species
